# Absence of Synergism between a Dual-AMP Biogel and Antibiotics Used as Therapeutic Agents for Diabetic Foot Infections

**DOI:** 10.3390/ijms25010407

**Published:** 2023-12-28

**Authors:** Rui Silva Soares, Diana Gomes, Isa Serrano, Eva Cunha, Luís Tavares, Manuela Oliveira

**Affiliations:** 1 CIISA—Center for Interdisciplinary Research in Animal Health, Faculty of Veterinary Medicine, University of Lisbon, Avenida da Universidade Técnica, 1300-477 Lisboa, Portugal; rmssoares@gmail.com (R.S.S.); dgomes@fmv.ulisboa.pt (D.G.); evacunha@fmv.ulisboa.pt (E.C.); ltavares@fmv.ulisboa.pt (L.T.); moliveira@fmv.ulisboa.pt (M.O.); 2 Associate Laboratory for Animal and Veterinary Sciences (AL4AnimalS), Avenida da Universidade Técnica, 1300-477 Lisboa, Portugal; 3 cE3c—Centre for Ecology, Evolution and Environmental Changes & CHANGE—Global Change and Sustainability Institute, Campo Grande 016, 1749-016 Lisbon, Portugal

**Keywords:** antimicrobial peptides, collagen model, nisin A, pexiganan, *Pseudomonas aeruginosa*, *Staphylococcus aureus*

## Abstract

Diabetic foot infections (DFIs) are frequently linked to diabetic-related morbidity and death because of the ineffectiveness of conventional antibiotics against multidrug-resistant bacteria. Pexiganan and nisin A are antimicrobial peptides (AMPs), and their application may complement conventional antibiotics in DFI treatment. A collagen 3D model, previously established to mimic a soft-tissue collagen matrix, was used to evaluate the antibacterial efficacy of a guar gum gel containing pexiganan and nisin alone and combined with three antimicrobials toward the biofilms of *Staphylococcus aureus* and *Pseudomonas aeruginosa* isolated from infected foot ulcers. Antimicrobials and bacterial diffusion were confirmed by spot-on-lawn and bacterial growth by bacterial count (cfu/mL). Our main conclusion was that the dual-AMP biogel combined with gentamicin, clindamycin, or vancomycin was not able to significantly reduce bacterial growth or eradicate *S. aureus* and *P. aeruginosa* DFI isolates. We further reported an antagonism between dual-AMP and dual-AMP combined with antibiotics against *S. aureus*.

## 1. Introduction

Diabetic foot ulcers (DFUs) are frequently associated with diabetic foot infections (DFIs). According to estimates, about 50% of patients living with diabetes with a DFU will eventually acquire a DFI [1]. Infected DFUs are the foremost reason for lower limb amputations [2] and are significantly more likely to worsen life quality in patients with diabetes, increase morbidity, and cause death [3].

DFIs are usually polymicrobial, with *Staphylococcus aureus* being the most common Gram-positive and *Pseudomonas aeruginosa* being the most common Gram-negative bacteria [4]. Both species have been reported to have increased resistance profiles to conventional antibiotics [5,6,7], compromising the efficacy of antimicrobial regimens used in DFI treatment. Therefore, it is urgent to seek alternative and more effective therapeutic approaches.

Antimicrobial peptides (AMPs) have been progressively explored as medicinal substitutes for traditional antibiotics in the treatment of microorganisms, including those on the WHO’s list of priority pathogens [8]. AMPs exhibit antimicrobial activity on pre-formed biofilms and prevent the formation of new ones [9]. Additionally, AMPs may act as immune modulators [8,10], exhibit low drug interaction, and have low toxicity [9,11], which suggests that they can be used in the therapy of foot ulcer infections.

Pexiganan and nisin are among the most thoroughly researched AMPs for alternative treatment strategies. Pexiganan is a synthesized AMP, similar to magainin, that acts by disrupting Gram-negative and Gram-positive bacteria’s cell membranes through toroidal-type pore formation [12]. At present, there are only 10 FDA-approved AMPs [13], and this list does not include pexiganan. After two phase III clinical trials, it was observed that pexiganan was not more effective than other available DFU treatments [14]. In a clinical study by Lipsky et al. (2008) [15], the effects of the topical administration of pexiganan and of the systemic administration of ofloxacin (a fluoroquinolone effectively used in the treatment of DFI) were compared [15], and the results showed that for mildly infected DFU, both drugs presented comparable clinic results. However, it was suggested that the topical application of pexiganan would be an alternative to the systemic administration of antimicrobials for the treatment of mildly infected foot ulcers, with the advantage that this AMP would avoid the development of resistance. The topical administration of pexiganan did not result in a noticeable improvement in comparison with the topical administration of a placebo [16,17]. Nevertheless, pexiganan’s extensive range of activity and reduced susceptibility to the development of resistance [18] justify its further research into the therapy of infected foot ulcers. Pexiganan is still under clinical investigation, and its approval may be achieved in the future [14]. Nisin A, a *Lactococcus lactis* peptide, exerts its antibacterial effect mostly against Gram-positive pathogens by inhibiting the incorporation of lipid II, the cell wall precursor, into the peptidoglycan network and by using it for subsequent pore formation [19]. Nisin A is efficient against staphylococci with multi-drug resistance in free [20,21] and biofilm forms [21,22,23], including those isolated from infected foot ulcers [24,25,26].

Numerous studies have established that the combination of different antibiotic compounds frequently increases their specific antimicrobial activity and widens their scope of activity [27], as observed with AMPs combined with other AMPs [28,29,30] or traditional antibiotics [29,31,32].

Recently, our team showed that the mixture of pexiganan and nisin A in a dual-AMP biogel delivery system can substantially reduce the pexiganan concentration required to inhibit free and biofilm forms of bacteria [28]. Moreover, it was shown that the combinatory use of these AMPs could eliminate *S. aureus* in a collagen three-dimensional model. However, in the same study, there was a limited inhibition of *P. aeruginosa* growth. This suggests that the association with other antibacterial agents might be advantageous to achieving *P. aeruginosa* eradication.

Different in vivo studies suggest that the treatment of some conditions, such as medical device infections [33] and sepsis [34], would benefit from the synergistic effects of AMPs combined with antimicrobials. Particularly, a combination of pexiganan and traditional antibiotics has been assessed in vitro against *S. aureus* [18,35,36] and *P. aeruginosa* [33] infections, as well as a combination of nisin and antibiotics against *S. aureus* [20,26,31,37] and *P. aeruginosa* [38] infections.

Broad-spectrum antibiotics commonly used in the treatment of DFI include, among others, dicloxacillin/flucloxacillin, amoxicillin-clavulanate, levofloxacin, cephalexin, ciprofloxacin, clindamycin, gentamicin, and vancomycin, and the choice of antibiotics to be applied depends on the clinical severity [2,39]. Topical therapy can be used in some mild superficial infections, but it is not advised for uninfected wounds [39]. Topical gentamicin is one of the most common administration routes of this antibiotic for DFI treatment [40,41,42]. We hypothesized that the combination of conventional antibiotics with a previously developed pexiganan-nisin dual-AMP biogel [28] would increase the efficacy of the biogel to be applied in the therapy of DFIs and other polymicrobial infections. The antibiotics selected for this study included clindamycin [43] and vancomycin [44], because they are usually active against community-associated Methicillin-resistant *Staphylococcus aureus* (MRSA) [39,44], and gentamycin, due to its activity against Gram-negative infections [45] and use in topical applications [40,41,42]. Therefore, in the present study, the antimicrobial efficacy of a pexiganan-nisin dual-AMP biogel combined with gentamycin, clindamycin, and vancomycin against *S. aureus* and *P. aeruginosa* was evaluated in a dual-species DFI collagen 3D model.

## 2. Results

### 2.1. Antimicrobial and Bacterial Diffusion in a DFI Collagen 3D Model

**Figure 1:**
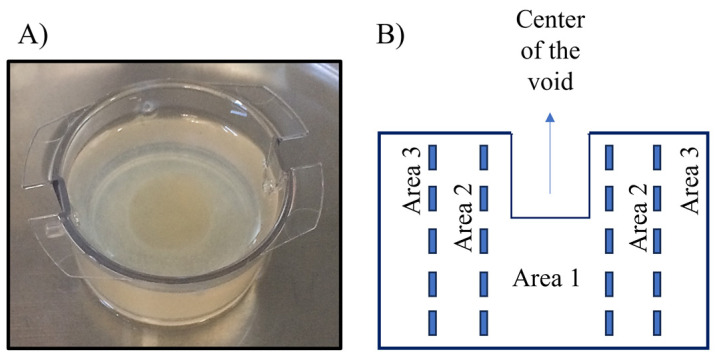
(**A**) Collagen 3D model; (**B**) Scheme of the collagen 3D model, showing area 1, 2 and 3 with increasing distances from the center of the void.

Antimicrobial and bacterial diffusion were assessed in a collagen 3D model (Figure 1).

**Figure 2:**
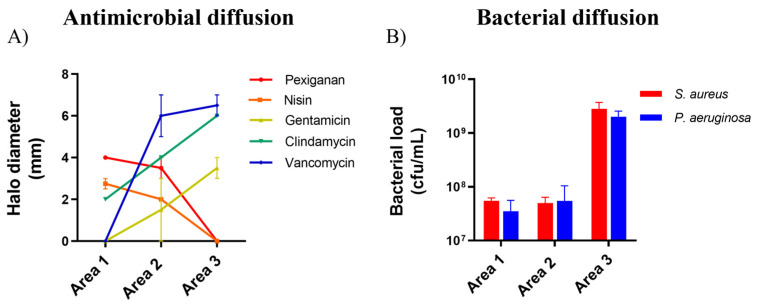
Antimicrobial and bacterial dispersal across areas 1, 2, and 3 of the collagen model. (**A**) Diffusion of pexiganan, nisin A, gentamicin, clindamycin, and vancomycin; (**B**) diffusion of *Pseudomonas aeruginosa* and *Staphylococcus aureus*. Data represent mean values ± standard deviation.

Both pexiganan and nisin diffused into Section 1 and Section 2 of the setup but were not identified in Section 3, as evidenced by the inhibitory halos formed by dual cultures of *S. aureus* Z25.2 and *P. aeruginosa* Z25.1 when inoculated with different sections of the setup (Figure 2A). In fact, pexiganan and nisin displayed higher inhibitory activity in area 1 than any of the antibiotics on the panel. In contrast, both gentamicin and vancomycin were not detected in area 1 but exhibited increasing inhibitory activity from area 2 to area 3, confirming diffusion through these areas. Of note, vancomycin was the antimicrobial from the panel that exhibited the highest inhibitory activity in the distal areas of the model (areas 2 and 3), and clindamycin was the only antimicrobial from the panel that exhibited antimicrobial activity simultaneously in all areas of the model (1, 2, and 3), 24 h after application, demonstrating that it was capable of diffusing and maintaining its antibacterial activity across the three areas. Clindamycin exhibited the second-highest antimicrobial activity in areas 2 and 3, and in area 1, it presented lower inhibitory activity than both pexiganan and nisin but higher than gentamicin and vancomycin (Figure 2A).

Both strains were also capable of diffusing through all sections of the setup, reaching similar concentrations (Figure 2B). Moreover, 24 h after inoculation, area 3 of the setup exhibited *S. aureus* and *P. aeruginosa* bacterial loads about 2-logs higher (2 to 3 × 10^9^ cfu/mL) than the bacterial load in area 1 or area 2 (3.5 to 5.5 × 10^7^ cfu/mL) (Figure 2B), demonstrating the ability of these strains to disperse over all sections of the 3D setup.

### 2.2. Inhibitory Activity of Pexiganan-Nisin Dual AMP Biogel Combined with Antibiotics

In the herein work, synergy was defined as a two-log cfu/mL reduction or greater achieved by the dual-AMP supplemented with antibiotics in comparison with the reduction promoted by the dual-AMP alone, and antagonism as a two-log reduction or greater achieved by the dual-AMP alone in comparison with the reduction promoted by the dual-AMP supplemented with antibiotics.

**Figure 3:**
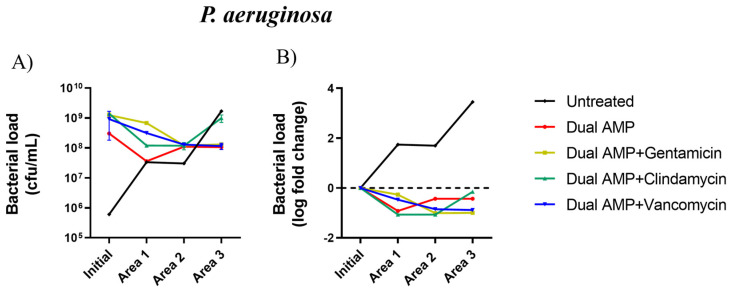
(**A**) *Pseudomonas aeruginosa* bacterial counts (cfu/mL) (mean values ± standard deviation); and (**B**) bacterial load log fold change (log fold change between bacterial load mean values) between initial inoculum (dashed line) and in the three areas of the model after treatment. AMP, antimicrobial peptide.

The use of the pexiganan-nisin biogel alone led to a 0.92 log decrease in *P. aeruginosa* bacterial load in area 1 and 0.44 log decrease in areas 2 and 3 (Table 1), as compared with the initial bacterial counts in these areas (3.0 × 10^8^ cfu/mL) (Figure 3A). In contrast, the association of gentamycin or vancomycin with the dual-AMP biogel resulted in a *P. aeruginosa* bacterial load decrease of 0.27 log and 0.47 log, respectively, in area 1 of the model and up to a 0.90 log decrease in the distal areas of the model (area 2 and area 3) (Table 1) in comparison with initial bacterial counts (1.3 × 10^8^ and 9.3 × 10^8^ cfu/mL) (Figure 3A). The association of clindamycin with the dual-AMP biogel led to a reduction of more than 1 log in *P. aeruginosa* bacterial load in areas 1 and 2, although only a 0.15 log reduction was observed in area 3 (Table 1), in comparison with initial bacterial counts in this model area (1.4 × 10^9^ cfu/mL) (Figure 3A). However, the *P. aeruginosa* bacterial load reductions achieved with either the dual-AMP biogel used alone or in combination with any of the antibiotics on the panel were not statistically different (*p* = 0.4487) (Figure 3A,B).

Concerning *P. aeruginosa*, the bacterial load reduction promoted by the dual-AMP alone in comparison with the one promoted by the dual-AMP gel supplemented with antibiotics was inferior to 2-log in the three areas, as their efficacy against this bacterium was similar (Table 1).

**Figure 4:**
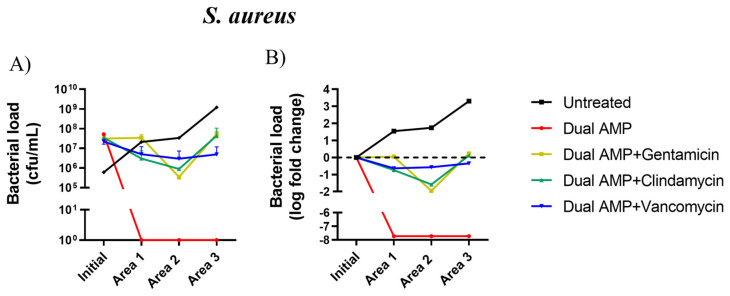
(**A**) *Staphylococcus aureus* bacterial counts (cfu/mL) (mean values ± standard deviation); and (**B**) bacterial load log fold change (log fold change between bacterial load mean values) between initial inoculum (dashed line) and in the three sections of the model after treatment. AMP, antimicrobial peptide.

Regarding *S. aureus*, the pexiganan-nisin gel alone could eliminate this bacterial species in the 3 sections of the DFI setup (100% decrease in microbial load), representing a 7.72 log reduction in bacterial load (Table 2, Figure 4A,B) in comparison with the initial bacterial load (5.2 × 10^7^ cfu/mL). The association of gentamycin with the AMPs gel led to a reduction in *S. aureus* load in area 2 of the model in comparison with the initial bacterial counts (3.2 × 10^7^ cfu/mL), representing a 1.95 log decrease in *S. aureus* load in this area of the model (Table 2, Figure 4A,B). However, in areas 1 and 3, this antimicrobial association was not able to inhibit *S. aureus* growth, resulting in a 0.04 and 0.23 log increase in bacterial load, respectively (Table 2, Figure 4A,B).

The association of clindamycin with the pexiganan-nisin gel led to a reduction in *S. aureus* load in Section 1 and Section 2, respectively, representing a 0.75 to 1.58 log reduction in bacterial load (Table 2, Figure 4A,B). However, a 0.10 log increase in bacterial counts was observed in area 3 (Table 2) in comparison with initial bacterial counts (3.4 × 10^7^ cfu/mL) (Figure 4A). The association of vancomycin with the AMPs gel reduced *S. aureus* bacterial counts in the three areas of the model. A 0.64, 0.56, and 0.35 log decrease in *S. aureus* counts were observed in areas 1, 2, and 3, respectively (Table 2, Figure 4A,B) in comparison with the initial *S. aureus* load (2.2 × 10^7^ cfu/mL). However, the *S. aureus* bacterial load reductions achieved with the pexiganan-nisin gel in combination with any antibiotics on the panel were not statistically different (*p* = 0.5099) (Figure 4A,B).

In *S. aureus*, the bacterial load reduction promoted by the dual-AMP alone in comparison with the one promoted by the dual-AMP gel supplemented with antibiotics was superior to 2-logs in the three areas, as the dual-AMP alone was more effective against this bacterium than when supplemented with antibiotics (Table 2), showing antagonistic behavior.

## 3. Discussion

Infections of foot ulcers are generally formed by polymicrobial biofilms [46]. In some cases, the use of conventional antimicrobial agents in therapeutic doses may not be sufficient, making the development of effective alternative treatments such as AMPs an important goal [47]. In the present study, a DFI collagen 3D model, previously developed by our group [28], was used to simulate the in vivo conditions of a foot ulcer. This setup allows biofilm development in a three-dimensional collagen matrix that replicates the poorly oxygenated foot ulcer environment. Nevertheless, it is important to note that the model is a closed system, and therefore, it does not account for other significant aspects that could happen in an infected foot ulcer, like the existence of immunity system cells and wound drainage. However, it is likely to be more accurate than alternative models using poly(methyl-methacrylate) (PMMA) or cellulose disks, as well as agar plate inhibition tests. Our data confirmed that the DFI collagen 3D model used in this study allows the effective diffusion of antimicrobials and bacteria, supporting its utility for the evaluation of new topical antimicrobial combinations aiming for effective control of polymicrobial DFIs. However, it is noteworthy to address the fact that the 2023 IWGDF/IDSA DFI guidelines suggest not using topical antibiotics in combination with systemic antibiotics, stating that more studies are needed to ensure the significant clinical usefulness of such treatments [48].

After establishing bacteria’s, AMPs, and antibiotics ability to diffuse through the DFI collagen 3D setup, it was found that the supplementation of the dual-AMP biogel with either gentamicin, clindamycin, or vancomycin was insufficient to promote the complete eradication of the two bacteria and that the dual-AMP gel combined with antimicrobials did not significantly decrease the microbial load of *S. aureus* and *P. aeruginosa*. Vancomycin acts through the inhibition of the peptidoglycans’ polymerization in the bacterial cell wall, whereas gentamicin and clindamycin inhibit the synthesis of proteins [49]. However, vancomycin and clindamycin are generally ineffective against Gram-negative bacteria and are unable to penetrate their outer membranes [43,44]. AMPs may improve antibiotic action by disrupting bacterial membranes and promoting their delivery, allowing antibiotics to act on intracellular targets [50]. Therefore, when antibiotics were combined with nisin and pexiganan, their inhibitory potential was expected to be improved, including the inhibition of *P. aeruginosa*’s growth. The absence of synergy between the inhibitory action of the dual-AMP and the antibiotics tested towards both *S. aureus* and *P. aeruginosa* was surprising since it has been described that the combinatory use of antimicrobials may exhibit synergistic effects, particularly when they have distinct action mechanisms [51], resulting in a potential antibacterial increment and an expanded action range [27]. This was previously reported by another study [52], in which the level of membrane disruption and permeabilization by different cationic-charged AMPs was not sufficient to drive them to act synergistically with antibiotics (ampicillin, ciprofloxacin, streptomycin, and vancomycin) against Gram-negative (*P. aeruginosa* and *Escherichia coli*) and Gram-positive (*S. aureus*) bacteria. Issues like differences in the time period in which pores promoted by the AMPs remain open, in their ability to inhibit pore repair, to disrupt bacterial intracellular processes, or to promote bacterial death by independent mechanisms [50], can eventually make the difference between the occurrence or absence of synergism. Probably, nisin and pexiganan take time to generate bacterial pores and do not prevent their resolution by cell repair. Optimized synergy testing techniques are needed to avoid bias and improve the use of antimicrobial combinations [53]. Still, the supplementation of the pexiganan-nisin gel with the antibiotics promoted an antibacterial increase against *P. aeruginosa* in some sections of the setup. As the model mimics soft tissue, one can infer that the use of the pexiganan-nisin gel combined with gentamicin or vancomycin would be useful to treat *P. aeruginosa* colonizing deep ulcers, and combined with clindamycin would be more suitable to treat *P. aeruginosa* colonizing superficial ulcers.

Our study confirmed that the dual-AMP biogel alone is able to eradicate *S. aureus*, as previously shown [28], achieving a bacterial load reduction of more than seven logs throughout the DFI 3D model. It is suggested that there is some form of antagonism when these antimicrobial agents are added to the dual-AMP gel against *S. aureus*. Antagonistic effects have been widely documented for specific antibacterial combinations and target species [54], including *S. aureus* [55,56]. There are multiple mechanisms that underlie antimicrobial interactions [57]. The antimicrobial activity of specific combinations of antimicrobial molecules is not only a function of their interactions but is also species-specific [57], making it challenging to determine ideal antimicrobial combinations in the context of polymicrobial infections. According to Brochado et al. [57], antagonism occurs almost exclusively between drugs with different mechanisms of action, like nisin-pexiganan vs. gentamicin, clindamycin, or vancomycin, being more common than synergy. Competitive binding to the same receptor or target is one of the reasons for antagonism.

The pore formation induced by the dual-AMP is believed to cause rapid dissipation of transmembrane electrostatic potential, leading to membrane permeabilization and consequent bacterial cell death [58,59]. In general, Gram-negative bacteria have a more negative surface electrical charge than Gram-positive ones due to the additional layer of negatively charged lipopolysaccharides [60]. These double protective membrane layers function as a barrier, blocking the action of cationic drugs, even though *P. aeruginosa*’s estimated negative surface charge may intensify their attraction. Therefore, like expected, the action of the dual-AMP promoted a higher cell death rate against *S. aureus* than against *P. aeruginosa*.

Unlike clindamycin, which is not charged, gentamicin and vancomycin are positively charged [61,62,63]; as such, when added to the dual-AMP, there may be a competition between the antimicrobials present in the biogel for the negatively charged bacterial membrane receptors, limiting pore formation by the AMPs, which is necessary for further antibiotic activity in intracellular targets. This competition may result in the antagonistic effect between the dual-AMP and the dual-AMP with antibiotics observed in *S. aureus*. In the case of *P. aeruginosa*, the double protective membrane layers present in this bacterial species are responsible for the inefficacy of the dual-AMP action, impeding the pore formation necessary for further antibiotic activity. Therefore, the effects of dual-AMP and dual-AMP combined with antibiotics on *P. aeruginosa* are neither antagonistic nor synergistic.

The fact that this study was based on a single *S. aureus* and *P. aeruginosa* DFI strain represents a limitation, and further research should evaluate combinations of the dual AMP biogel with other antibiotics or AMPs to take full advantage of the pexiganan-nisin gel’s positive prospects for DFI treatment.

## 4. Materials and Methods

### 4.1. Bacterial Isolates

*Staphylococcus aureus* Z25.2 and *Pseudomonas aeruginosa* Z25.1 are biofilm-producer strains and were isolated from the same infected foot ulcer. These isolates were chosen from a bacterial pool collected from patients with infected DFUs [4] and previously characterized [64]. Both isolates were kept and grown as described [28]. Briefly, isolates kept in buffered peptone water with 20% (*v*/*v*) of glycerol were stored at −80 °C. When necessary, they were inoculated on Brain Heart Infusion medium (VWR Chemicals, Leuven, Belgium) and then incubated for 24 h at 37 °C. Dual-microbial suspensions were made with equivalent amounts of each bacterium.

### 4.2. Antibiotics and Antimicrobial Peptides

Stock solutions of gentamicin (ITW Reagents, Monza, Italy) were prepared at 4760 µg/mL, clindamycin (Cayman Chemical, Michigan, MI, USA) at 660 µg/mL, and vancomycin (ITW Reagents, Monza, Italy) at 1062 µg/mL, according to the manufacturer’s specifications, and kept at −80 °C. Antibiotic working solutions were used at their minimum inhibitory concentration (MIC) values (gentamicin, 0.238 µg/mL; clindamycin, 0.033 µg/mL; and vancomycin, 0.531 µg/mL), as determined in a previous study [65].

Nisin A (1000 UI/mg, 2.5% purity) (Sigma-Aldrich, St. Louis, MO, USA) was dissolved in 0.02 M HCl (Merck, Darmstadt, Germany), yielding a 1000 µg/mL stock solution, and pexiganan (>95% purity; Innovagen, Lund, Sweden) was dissolved in deionized sterile water, yielding a 2048 µg/mL stock solution. These solutions were then filtered through a 0.22 µm filter (Millipore Corporation, Billerica, MA, USA) and kept at 4 °C. To prepare the dual-AMP biogel, a pexiganan solution at 256 µg/mL was enriched with nisin at 125 µg/mL, corresponding to their minimum biofilm eradication concentration (MBEC) values, as determined previously [25]. 0.75 g of guar gum (Sigma-Aldrich, St. Louis, MO, USA) was dissolved in 50 mL of sterile distilled water, yielding a 1.5% (*w*/*v*) gel, which was sterilized by autoclave. Antibiotics and AMPs were mixed into the gel in a 1:1 ratio.

### 4.3. Collagen Model

The susceptibility of *S. aureus* and *P. aeruginosa* to the dual-AMP biogel combined with antibiotics was assessed in a 3D structure similar to the collagen matrix found in soft tissues. The setting of the DFI model was adapted from prior experiments [66,67] and assembled as described [28]. Briefly, the setting comprised 6 well plates and respective transwells with 3.0 μm pore polyester membrane inserts (Corning Incorporated, Corning, NY, USA). The collagen solution comprising 25% (*v*/*v*) of rat tail Collagen I at 8.24 mg/mL (Corning Incorporated, Corning, NY, USA), 25% of NaOH at 0.1M (Merck, Darmstadt, Germany), and 50% (*v*/*v*) of cold Simulated Wound Fluid (SWF), adjusted to a pH of 7.5, was polymerized within the inserts that served as mold. The SWF was prepared by combining peptone water (Biokar Diagnostics, Allone, France) and fetal bovine serum (Biowest, Nuaillé, France) in a 1:1 ratio. To create the collagen inserts, each one was loaded with 8 mL of collagen solution, creating an insert with a 28 mm diameter and 15 mm depth. Afterwards, plates were covered by a sterilized peg-lid, which was used as a mold to form voids (12 mm in diameter and 5 mm in depth) in the collagen inserts, mirroring the dimensions of an ulcer of grade 1B [68]. Finally, the setup was kept for 90 min at 37 °C to allow the collagen to polymerize in a humid chamber (Figure 1A).

### 4.4. Antimicrobials and Bacterial Diffusion Using the Collagen Model

The AMPs and antibiotics diffusion across the collagen model was confirmed using a procedure previously described [28]. Briefly, 5 mL of SWF were added to collagen inserts, and each void was filled with 2 mL of antimicrobials (pexiganan, nisin, gentamicin, clindamycin, or vancomycin). Then, the setting was incubated for 24 h at 37 °C. Antimicrobial diffusion was assessed through the quantification of the antimicrobial activity of compounds present in three different sections of the 3D setup, representing increasing distances from the center of the void (area 1 corresponds to the inner circle with 1.5 cm diameter; area 2 corresponds to 2.0 cm diameter; area 3, >2 cm diameter) (Figure 1B). For that purpose, a collagenase solution at 500 μg/mL in PBS (Merck, Darmstadt, Germany) was used to digest the collagen from each section for 90 min at 37 °C. The supernatants from each digestion were extracted by centrifugation (4000× *g*, 10 min, 4 °C). After that, 20 μL of each supernatant was added to trypticase soy agar (TSA) plates, fully covered by 1 × 10^7^ cfu/mL of *P. aeruginosa* and *S. aureus,* and kept for 24 h at 37 °C. Antimicrobial activity in each area of the model was assessed by measuring the inhibition halos formed in the presence of each antimicrobial solution.

To confirm bacterial diffusion, the wells were filled with 5 mL of SWF, together with 500 μL of a dual suspension of *P. aeruginosa* and *S. aureus* prepared in SWF at 1 × 10^6^ cfu/mL each, and incubated for 24 h at 37 °C. After that, the collagen setup was split, digested, and pelleted as indicated above. Each pellet was resuspended in saline solution, serially diluted 10 times, and 100 μL were added to TSA plates and placed in the incubator for 24 h at 37 °C for subsequent colony counting. The tests were carried out in duplicate.

### 4.5. Assessment of the Antimicrobial Activity of the Gel

To assess the inhibition ability of the dual-AMP biogel in combination with the conventional antibiotics, 5 mL of SWF and 500 μL of a dual suspension of *P. aeruginosa* and *S. aureus* at 1 × 10^6^ cfu/mL each were applied to each well. Subsequently, the 6-well plate was placed in a humid chamber at 37 °C for 24 h to let microorganisms disperse. The insert was then filled with 2 mL of each antibacterial solution, composed of a biogel of guar-gum supplemented with both nisin and pexiganan (dual-AMP gel) or a dual-AMP gel supplemented with one of the selected antibiotics, and incubated in a humid environment for 8 h at 37 °C. Afterwards, 2 mL of the inoculated SWF present in the well were removed and replaced by 2 mL of antibacterial solution, and the plate was again incubated. This procedure was performed three times for 24 h. Then, microbial quantification was carried out in duplicate, as described in Section 4.4.

### 4.6. Statistical Analysis

GraphPad Prism 5 for Windows was used for statistical evaluation. Differences between bacterial loads in the different sections of the collagen model after treatment with pexiganan-nisin biogel alone and treatment with the different antimicrobial combinations were determined by one-way ANOVA analysis of variance, followed by Dunnet’s post-test. Statistical significance was defined as a two-tailed *p* value ≤ 0.05.

## 5. Conclusions

This study showed that the dual-AMP biogel used in combination with gentamicin, clindamycin, or vancomycin was not able to significantly reduce bacterial growth nor eradicate *S. aureus* and *P. aeruginosa* biofilms in a collagen 3D setup. The biogels composed of the dual-AMP and the dual-AMP combined with antibiotics exhibited antagonistic inhibitory behavior against *S. aureus*, as the dual-AMP biogel alone was more effective against this bacterium than when supplemented with antibiotics.

## Figures and Tables

**Table 1:** *Pseudomonas aeruginosa* load reduction (log) in the three areas of the collagen 3D model (in comparison with the initial bacterial load mean values) after treatment with the dual-antimicrobial peptide (AMP) solution alone or supplemented with either vancomycin, clindamycin, or gentamicin; and bacterial load reduction (log) promoted by the dual-AMP alone in comparison with the dual-AMP supplemented with antibiotics in each area.

Compounds	Area 1		Area 2		Area 3	
	Log Load Reduction	Log Load Reduction of the Dual AMP Alone	Log Load Reduction	Log Load Reduction of the Dual AMP Alone	Log Load Reduction	Log Load Reduction of the Dual AMP Alone
Dual-AMP	−0.92		−0.44		−0.44	
Dual-AMP + gentamicin	−0.27	−1.28	−1.01	−0.04	−0.98	−0.08
Dual-AMP + clindamycin	−1.07	−0.52	−1.07	−0.04	−0.15	−0.96
Dual-AMP + vancomycin	−0.47	−0.94	−0.85	−0.07	−0.90	−0.02

**Table 2:** *Staphylococcus aureus* load reduction (log) in the three areas of the collagen 3D model (in comparison with the initial bacterial load mean values) after treatment with the dual-antimicrobial peptide (AMP) solution alone or supplemented with either vancomycin, clindamycin, or gentamicin; and bacterial load reduction (log) promoted by the dual-AMP alone in comparison with the dual-AMP with antibiotics in each area.

Compounds	Area 1		Area 2		Area 3	
	Log Load Reduction	Log Load Reduction of the Dual AMP Alone	Log Load Reduction	Log Load Reduction of the Dual AMP Alone	Log Load Reduction	Log Load Reduction of the Dual AMP Alone
Dual-AMP	−7.72		−7.72		−7.72	
Dual-AMP + gentamicin	0.04	−7.54 *	−1.95	−5.54 *	0.23	−7.73 *
Dual-AMP + clindamycin	−0.75	−6.79 *	−1.58	−5.95 *	0.10	−7.64 *
Dual-AMP + vancomycin	−0.64	−6.70 *	−0.56	−6.78 *	−0.35	−7.00 *

* Antagonism: Dual-AMP showing a bacterial load reduction greater than 2-log in comparison with dual-AMP with antibiotics.

## Data Availability

The data presented in this study are available on request from the corresponding author. The data are not publicly available due to privacy reasons.
